# Relationship between the Antifungal Susceptibility Profile and the Production of Virulence-Related Hydrolytic Enzymes in Brazilian Clinical Strains of *Candida glabrata*

**DOI:** 10.1155/2017/8952878

**Published:** 2017-07-26

**Authors:** Maria Helena Galdino Figueiredo-Carvalho, Lívia de Souza Ramos, Leonardo Silva Barbedo, Jean Carlos Almeida de Oliveira, André Luis Souza dos Santos, Rodrigo Almeida-Paes, Rosely Maria Zancopé-Oliveira

**Affiliations:** ^1^Laboratório de Micologia, Instituto Nacional de Infectologia Evandro Chagas, Fundação Oswaldo Cruz, Rio de Janeiro, RJ, Brazil; ^2^Laboratório de Investigação de Peptidases, Departamento de Microbiologia Geral, Instituto de Microbiologia Paulo de Góes, Universidade Federal do Rio de Janeiro, Rio de Janeiro, RJ, Brazil

## Abstract

*Candida glabrata* is a facultative intracellular opportunistic fungal pathogen in human infections. Several virulence-associated attributes are involved in its pathogenesis, host-pathogen interactions, modulation of host immune defenses, and regulation of antifungal drug resistance. This study evaluated the in vitro antifungal susceptibility profile to five antifungal agents, the production of seven hydrolytic enzymes related to virulence, and the relationship between these phenotypes in 91 clinical strains of *C. glabrata*. All *C. glabrata* strains were susceptible to flucytosine. However, some of these strains showed resistance to amphotericin B (9.9%), fluconazole (15.4%), itraconazole (5.5%), or micafungin (15.4%). Overall, *C. glabrata* strains were good producers of catalase, aspartic protease, esterase, phytase, and hemolysin. However, caseinase and phospholipase in vitro activities were not detected. Statistically significant correlations were identified between micafungin minimum inhibitory concentration (MIC) and esterase production, between fluconazole and micafungin MIC and hemolytic activity, and between amphotericin B MIC and phytase production. These results contribute to clarify some of the *C. glabrata* mechanisms of pathogenicity. Moreover, the association between some virulence attributes and the regulation of antifungal resistance encourage the development of new therapeutic strategies involving virulence mechanisms as potential targets for effective antifungal drug development for the treatment of *C. glabrata* infections.

## 1. Introduction

*Candida glabrata* is a facultative intracellular opportunistic fungal pathogen, with the ability to survive and replicate in several cell types, such as osteoblasts [1], neutrophils [2], and macrophages [3]. This yeast can be isolated from different areas of the human body such as mouth, gastrointestinal tract, and vaginal mucosa, without causing disease in most individuals [4]. Nevertheless, due to the increased use of immunosuppressive drugs and the advent of AIDS, the frequency of *C. glabrata* infections has significantly increased worldwide in the last years [5–8].

In the last decade, two new species phenotypically related to *C. glabrata* have been described in the literature: *Candida nivariensis* and *Candida bracarensis.* These three species are phenotypically indistinguishable, but genetically heterogeneous [9, 10]. It is necessary to periodically monitor the *C. glabrata* species complex in order to determine the frequency of these clinically relevant *Candida* species, their geographical distribution, their virulence attributes, and their propensity to harbor antifungal resistance mechanisms [11, 12].

The therapeutic and prophylactic use of azole antifungals administered for prolonged periods to invasive candidiasis treatment, especially in immunocompromised patients, has contributed to the increase phenomenon of resistance in *C. glabrata* [5, 7, 13]. The echinocandins have emerged as preferred agents for most episodes of candidemia and invasive candidiasis according to the recent guideline for the management of candidiasis [14]. Nevertheless, echinocandin resistance is increasing in *C. glabrata* [15], including among fluconazole-resistant isolates [5, 15, 16].

The pathogenicity of *Candida* spp. is facilitated by expression on several virulence-associated factors, especially the adherence to host cells, the ability to form biofilms, the resistance to hydrogen peroxide and derivatives, and the capacity to produce and secrete hydrolytic enzymes, particularly proteases, phospholipases, and hemolysins [17, 18]. In comparison with *C. albicans,* there are fewer studies about the potential virulence attributes produced by *C. glabrata*.

The present study aimed to evaluate the in vitro antifungal susceptibility profile, the production of hydrolytic enzymes, and the relationship between these phenotypes in a collection of *C. glabrata* clinical strains isolated from Brazilian hospitals.

## 2. Materials and Methods

### 2.1. Fungal Strains

A total of 91 yeast strains, collected between 1998 and 2015 in two tertiary hospitals located in Rio de Janeiro, Brazil, and preliminarily identified by the API 20C AUX (bioMérieux, France) as *C. glabrata*, were included in this study. Strains were isolated from several clinical specimens, such as gastric aspirate (*n* = 1); renal abscess secretion (*n* = 1); pleural fluid (*n* = 1); secretion of surgical drain (*n* = 1); secretion of postoperative wound (*n* = 1); ascitic fluid (*n* = 2); abdominal secretion (*n* = 3); peritoneal fluid (*n* = 4); sputum (*n* = 4); venous catheter (*n* = 4); bronchoalveolar lavage (*n* = 5); vaginal secretion (*n* = 7); feces (*n* = 9); tracheal secretion (*n* = 10); urine (*n* = 13); and blood (*n* = 25). Before the experiments, these clinical strains were recovered from storage (−20°C) and grown on Sabouraud Dextrose Agar and CHROMagar *Candida* medium (both at 37°C for 48 h) in order to evaluate their viability and purity, respectively. The phenotypic confirmation of the species after storage was achieved by a biochemical analysis with the Vitek 2 system (bioMérieux, Marcy-L'Etoile, France) using the YST card according to the manufacturer's guidelines. In addition, *C. glabrata* ATCC 2001 type strain was included as a control strain in all experiments.

### 2.2. Molecular Identification

Yeast cells obtained from pure colonies were recovered from Sabouraud Dextrose Agar and used for DNA extraction with the Gentra® Puregene® Yeast and G+ Bacteria Kit (Qiagen®). The strains were identified by sequencing the ITS1-5.8S-ITS2 region of the rDNA as previously described [9], using the primers ITS1 (5′-TCCGTAGGTGAACCTGCGG-3′) and ITS4 (5′-TCCTCCGCTTATTGATATGC-3′). Sequences were edited using the Sequencher™ version 4.9 and compared by BLAST with sequences available from the NCBI/GenBank database.

### 2.3. Antifungal Susceptibility Testing

In vitro antifungal susceptibility testing was performed according to the recommendations proposed by the Clinical and Laboratory Standards Institute (CLSI) M27-A3 protocol [19]. Amphotericin B (AMB), fluconazole (FLC), itraconazole (ITC), micafungin (MCF), and 5-flucytosine (5-FC) (Sigma-Aldrich Chemical Corporation, St. Louis, MO, USA) were tested. Briefly, RPMI 1640 medium with L-glutamine and without bicarbonate (Gibco BRL, Life Technologies, Woerden, The Netherlands), buffered with 0.165 M 3-*N-*morpholinepropanesulfonic acid (MOPS), pH 7.0, was used for the broth microdilution test. Two-fold dilutions of the drugs were performed and distributed in 96-well flat bottom plates in concentrations ranging from 64–0.125 *μ*g/mL for FLC and 5-FC, 8–0.015 *μ*g/mL for AMB and ITC, or 4–0.008 *μ*g/mL for MCF. The fungal inoculum was prepared from a 24 h Sabouraud Dextrose Agar culture incubated at 35°C; the cells were harvested in RPMI medium and diluted to about 1–5 × 10^3^ cells/mL. The plates were incubated at 35°C for 24 h. The minimal inhibitory concentrations (MIC) of the drugs were determined according to the CLSI M27-A3 recommendations [19]; and the MIC values for AMB, ITC, and 5-FC were interpreted following the CLSI M27-S3 protocol; and the MIC values for FLC and MCF were interpreted according to the CLSI M27-S4 protocol [20, 21]. MICs were validated after a second experiment performed under the same conditions with the same MIC value verified for each strain.

### 2.4. Production of Hydrolytic Enzymes

The production of hydrolytic enzymes was carried out in agar plate assays as described previously by Price et al. [22]. Briefly, the aspartic protease activity was determined using 1.17% yeast carbon base medium supplemented with 0.2% bovine serum albumin according to Rüchel et al. [23]. Caseinase activity was assessed using Sabouraud Dextrose Agar provided with 1% casein as previously described by Ziccardi et al. [24]. The determination of phospholipase activity was performed using the egg yolk agar plate method (2% glucose, 1% peptone, 0.5% yeast extract, 4% NaCl, 0.074% CaCl_2_, 1.5% agar, then, 2% of fresh egg yolk was added to the medium) as previously described by Price et al. [22]. The esterase production was assayed using the Tween agar plate (0.5% yeast extract, 1% peptone, 0.01% CaCl_2_, 1.5% agar, and 0.1% Tween 80, pH 7.0) according to Aktas et al. [25]. Phytase activity was evaluated using the calcium phytate agar (1% glucose, 0.05% (NH_4_)_2_SO_4_, 0.02% KCl, 0.01% MgSO_4_·7H_2_O, 0.2% calcium phytate, 0.05% yeast extract, 0.0005% MnSO_4_, 0.0005% FeSO_4_, and 1.5% agar, pH 7.0) according to Tsang [26]. The hemolytic activity was evaluated in a commercial blood agar plate assay (Plast Labor, Brazil). To determine enzymatic activities, aliquots (10 *μ*l) of 48 h old cultured fungal cells (10^7^ cells) were spotted on the surface of each agar medium and incubated at 37°C for up to 7 days. The colony diameter (*a*) and the diameter of the colony plus the precipitation zone (*b*) were measured by a graduated ruler, and the enzymatic activities were expressed as *Pz* value (*a/b*) as previously described [22]. The *Pz* value was scored into four categories: *Pz* of 1.0 indicated no enzymatic activity; *Pz* between 0.999 and 0.700 indicated weak producers; *Pz* between 0.699 and 0.400 corresponded to good producers; and *Pz* lower than 0.399 meant excellent producers [22].

Determination of catalase activity was performed using a semiquantitative assay with slight modifications according to Metchock et al. [27]. In brief, screw-cap tubes containing Sabouraud Dextrose Agar medium were inoculated with 200 *μ*L of a suspension of *C. glabrata* cells corresponding to the 0.5 McFarland standard and incubated at 37°C for 48 h. After this incubation, 1 mL of a freshly prepared 1 : 1 mixture of 10% Tween 80 and 30% hydrogen peroxide was added to the cultures. The column bubble was measured in millimeters after 5 min at room temperature. Uninoculated medium was used as a negative control. A column of bubbles of <45 mm was classified as low catalase producers, while a column bubble of >45 mm was classified as high catalase producers [27].

Since media and conditions may play a key role in the gene expression of the enzymes studied, all enzymatic tests were performed with culture media prepared from a single bottle and tested using the same equipment. Moreover, all the tests for the determination of production of hydrolytic enzymes were performed in duplicate, and results of enzymatic activities are presented as mean ± standard deviation (SD).

### 2.5. Statistical Analysis

The statistical analyses were performed with the GraphPad Prism 5 computer software®. The correlation between MIC values and the enzymatic activity was performed using the Spearman's rank correlation, since the variables do not meet the bivariate normal distribution assumption. The strength of the relationship between paired data was interpreted through the Spearman's correlation coefficient (*r_s_*) analysis, where the closer *r_s_* is to ±1, the stronger the relationship. Additionally, strains were grouped according to their susceptibility profile (susceptible-dose dependent/resistant for FLC; susceptible/nonsusceptible for other drugs), and the median value of the enzymatic activity of each group was compared using the Mann–Whitney *U* test. *P* values of 0.05 or less were considered to be statistically significant in all tests.

## 3. Results

### 3.1. Phenotypic and Molecular Identification of Fungal Strains

All the 91 clinical yeast strains produced colonies with a coppery pigment and smooth texture on chromogenic CHROMagar *Candida* medium, and contamination or mixed colonies were not detected. According to the biochemical analysis by the Vitek 2 system, these strains were identified as *C. glabrata* with an average probability of 98%.

Moreover, all the 91 yeast strains were identified through sequencing of ITS1-5.8S-ITS2 region of the rDNA. These clinical strains showed 99-100% similarity when compared to the *C. glabrata* AY939793 sequence deposited in the GenBank database, thus confirming their identity as *C. glabrata*. No *C. nivariensis* or *C. bracarensis* was found in this study. The obtained sequences to ITS1-5.8S-ITS2 region of the clinical strains were deposited in GenBank under the accession numbers KX450781-KX450814, KX450816-KX450833, KX450835-KX450861, and KX450863-KX450874.

### 3.2. Susceptibility of *C. glabrata* against Five Antifungal Drugs

Concerning the antifungal susceptibility profile (Table 1), all the 91 clinical strains of *C. glabrata* were susceptible to 5-FC. However, some of these strains showed resistance to AMB, FLC, ITC, or MCF. In brief, nine *C. glabrata* strains (9.9%) were likely to be resistant to AMB as follows: five strains exhibited MIC of 2 *μ*g/mL, one strain presented MIC of 4 *μ*g/mL, and three strains exhibited MIC of 8 *μ*g/ml to this polyene agent. FLC was the azole with the highest number of resistant strains (MIC ≥ 64 *μ*g/mL). A total of 14 strains (15.4%) were resistant to FLC, whereas five (5.5%) presented resistance to ITC. Fourteen strains of *C. glabrata* (15.4%) exhibited MIC > 0.12 *μ*g/mL to MCF.

The *C. glabrata* ATCC 2001 type strain was classified as susceptible-dose dependent to FLC (MIC of 8 *μ*g/mL) and susceptible to AMB, ITC, MCF, and 5-FC (MIC of 0.12, 0.06, 0.06, and 0.12 *μ*g/mL, resp.).

Eleven of the 91 strains tested (12.1%) were classified as resistant to at least two antifungal drugs.  Table 2 summarizes the resistance profile of the Brazilian tested clinical strains of *C. glabrata*.

Association between resistance and the clinical origin of strains or year of isolation was not detected for any of the tested antifungal drugs (*P* > 0.05).

### 3.3. Production of Hydrolytic Enzymes

In this set of experiments, the in vitro abilities of the *C. glabrata* to produce proteases (aspartic protease and caseinase), phospholipase, esterase, phytase, hemolysin, and catalase were evaluated. Phospholipase and caseinase activities were not detected under the employed experimental conditions for any of the tested strains. Eighty-seven strains of *C. glabrata* (95.6%) were able to produce aspartic protease (*Pz* ranging from 0.100 to 0.583), while four strains (4.4%) showed no enzymatic activity for this hydrolytic enzyme (*Pz =* 1.0). The clinical strains of *C. glabrata* producing aspartic protease were classified as follows: 30 clinical strains (33.0%) were considered excellent producers (*Pz* ranging from 0.100 to 0.395), and 57 clinical strains (62.6%) were classified as good producers (*Pz* ranging from 0.400 to 0.583).

Esterase was detected in 51 *C. glabrata* strains (56.0%), being one strain (1.1%) classified as excellent esterase producer (*Pz* mean = 0.393 ± 0.050), 48 strains (52.7%) were considered good producers (*Pz* ranging from 0.414 to 0.667), and two strains (2.2%) were considered weak producers (*Pz* ranging from 0.762 to 0.800).

Regarding the phytase production, all the strains were positive (*Pz* ranging from 0.114 to 0.762), in which 10 strains (11.0%) were considered excellent producers (*Pz* ranging from 0.114 to 0.380), 80 strains (87.9%) were classified as good producers (*Pz* ranging from 0.400 to 0.692), and one strain (1.1%) was considered weak phytase producer (*Pz* mean = 0.762 ± 0.050).

Hemolytic activity was observed in 90 *C. glabrata* strains (98.9%), being one strain (1.1%) considered excellent producer of hemolysins (*Pz* mean = 0.385 ± 0.000), 82 strains (90.1%) classified as good producers (*Pz* ranging from 0.409 to 0.688), and seven strains (7.7%) were considered weak producers (*Pz* ranging from 0.722 to 0.795).

The *C. glabrata* ATCC 2001 type strain was considered an excellent aspartic protease producer (*Pz* mean = 0.357 ± 0.034) and a weak producer of phytase (*Pz* mean = 0.714 ± 0.000). Caseinase, phospholipase, esterase, and hemolytic activities were not detected under the employed experimental conditions for this strain.

The activity of catalase was detected in all *C. glabrata* strains studied, including *C. glabrata* ATCC 2001 type strain. All the strains produced bubbles almost immediately after hydrogen peroxide hydrolysis, and these strains were classified as high catalase producers.

The profile of hydrolytic enzymes related to virulence of the strains was not related to the clinical origin of the strains nor the year of strain isolation (*P* > 0.05).

### 3.4. Relationship between Antifungal Susceptibility Profile and Virulence Attributes

Spearman's correlation revealed significant associations between phytase production and AMB MIC, hemolysin production and FLC MIC, esterase production and MCF MIC, and hemolysin production and MCF MIC (Table 3). According to the *r_s_* analysis, phyatse *Pz* and AMB MIC, hemolysin *Pz* and FLC MIC, hemolysin *Pz* and MFC MIC have a negative monotonic correlation, whereas esterase *Pz* and MCF MIC are positively monotonically correlated. Moreover, the strength of all negatively correlated variables was classified as weak, and the esterase/MCF correlation was classified as moderate.

**Figure 1:**
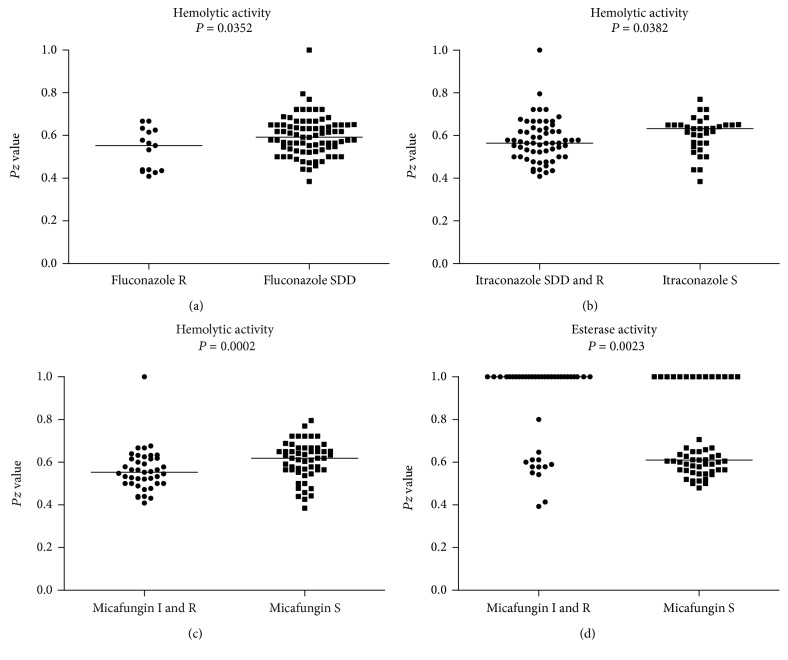
Differential expression of hydrolytic enzymes by 91 *Candida glabrata* strains with different susceptibility profiles against antifungal drugs: (a) hemolytic activity of strains regarding fluconazole susceptibility; (b) hemolytic activity of strains regarding itraconazole susceptibility; (c) hemolytic activity of strains regarding micafungin susceptibility; and (d) esterase activity of strains regarding micafungin susceptibility. Straight lines in each group represent the median for that group. In all the cases, differences between medians of groups with different susceptibility profiles were statistically significant (*P* < 0.05).

Regarding the enzymatic activities of strains grouped according to their susceptibility profile, differences in the median production value of all studied hydrolytic enzymes were not detected in strains of *C. glabrata* with different susceptibilities to AMB (*P* > 0.05). However, statistically significant differences on the median esterase *Pz* values were noticed between strains with different MCF susceptibility profiles and also on the median *Pz* values for hemolytic activity between strains with different FLC, ITC, and MCF susceptibility profiles (Figure 1).

## 4. Discussion

Phenotypic methods are not able to discriminate among *C. glabrata*, *C. nivariensis*, and *C. bracarensis* [9, 10]. Therefore, as suggested by others authors [9, 28], a molecular method based on sequencing of ITS1-5.8S-ITS2 region of the rDNA was employed to conclude the identification of the clinical strains analyzed in this study. *C. glabrata* was the sole species found. These results are in agreement with the previous studies [12, 29], showing the high prevalence of *C. glabrata* taken into consideration the *C. glabrata* species complex. The correct identification of yeast species causing invasive mycoses is fundamental to ensure proper management of the patient and specific, early, and effective antifungal therapy [9, 30, 31].

Among the antifungal agents used in the management of candidiasis, we can highlight the amphotericin B, fluconazole, itraconazole, voriconazole, posaconazole, isavuconazole, echinocandins, and 5-flucytosine [14]. Unfortunately, only FLC and the echinocandins have clinical breakpoints described by the CLSI to *C. glabrata* [21]. Although no clinical breakpoints for AMB have been suggested, the CLSI document indicates that MIC values for this antifungal drug higher than 1 *μ*g/mL are suggestive of resistance [19], the reason for the inclusion of this drug in our analysis. Clinical breakpoints for caspofungin and *C. glabrata* have been described. However, some studies have pointed that the broth microdilution testing is not suitable for caspofungin MIC determination, since unexplained interlaboratory differences are very common for this drug [32–34], and therefore caspofungin was not included in this study. Instead, MCF was chosen to check whether virulence attributes regulate echinocandins' resistance, since this drug does not raise the same problems observed during MIC determination of caspofungin [34].

In this study, the majority of *C. glabrata* strains presented a MIC ≤ 1 *μ*g/mL to AMB. Fluconazole and micafungin resistance were noted among some *C. glabrata* strains. Similar results were observed in a Portuguese multicenter survey [35] and in a global study developed during the 2014 SENTRY antifungal surveillance program. [36]. However, previous studies developed in Peru [37] and Brazil [38], with a small number of strains (*N* = 8 and 15 isolates, resp.) did not find *C. glabrata* strains with AMB MIC > 1 *μ*g/mL.

According to the clinical breakpoints for *C. glabrata*, it was observed that the frequency of resistant strains was higher to FLC and MCF. Moreover, some *C. glabrata* strains were resistant to both FLC and MCF. Similar results were found in other studies showing that fluconazole-resistant *C. glabrata* isolates were resistant to one or more echinocandins [5, 15]. Echinocandins' resistance appears to be associated with prior exposure to these drugs as well as the presence of *FKS* mutations [15, 16], while azole's resistance can be the result of an alteration of the lanosterol 14*α*-demethylase target enzyme by either overexpression or mutations in its encoding gene *ERG11* [39], or overexpression of efflux pumps mediated by the activation of expression of ATP-binding cassette (ABC) or major facilitator superfamily (MFS) transporters [40–42].

In this study, flucytosine demonstrates the greatest in vitro antifungal activity against *C. glabrata* clinical strains. However, in vivo, this drug is usually given in combination with another antifungal agent due to a high rate emergence of resistance during monotherapy for candidiasis [14].

In addition to the CLSI method employed in this study, the only other international standard method for antifungal susceptibility testing of yeasts is that published by European Committee on Antimicrobial Susceptibility Testing (EUCAST) [43]. Pfaller et al. [44] compared these two standardized methods for 10 antifungal agents, including amphotericin B, fluconazole, itraconazole, micafungin, and flucytosine against a collection of clinical isolates of *Candida albicans*, *C. glabrata*, *C. parapsilosis*, *C. tropicalis*, and *C. krusei*. The results indicate that the CLSI and EUCAST methods produce similar results for antifungal susceptibility testing against the five most common species of *Candida*, indicating that their use should not result in resistance profiles different enough to affect direct treatment decisions.

In *Candida* species, extracellular hydrolytic enzymes facilitate the nutrition, adherence, colonization, penetration of tissues or cells, invasion, dissemination, and escape from host immune responses [18, 45]. Moreover, secretion of hydrolytic enzymes has the ability to regulate *Candida* spp. antifungal drug resistance [46].

Aspartic proteases are enzymes with high proteolytic activity and stability at acid pH [47]. These enzymes control several steps in innate immune evasion, and they degrade proteins related to immunological defense such as antibodies, complement, and citokines, allowing the fungus to escape from the first line of host defenses [48]. Moreover, a study developed by Silva et al. [46] suggests that naturally resistant *Candida* spp. or isolates that have developed resistance after prolonged exposure to drugs may present an increase in the secretion pattern and proteolytic activity of secreted aspartic proteases (SAP), but more studies are needed to elucidate its relation. In our study, most strains of *C. glabrata* were classified as good aspartic protease producers. However, *C. glabrata* does not possess classical *SAP* genes in its genome [46, 49]. Probably the enzymatic degradation of albumin verified herein may be due to the production of yapsins. The yapsins (YPS) are a family of five nonsecreted glycosylphosphatidyinositol-linked aspartic proteases that have a well-known role in cell wall integrity and increase the capacity of the fungus to survive inside human macrophages [50]. A study developed by Swoboda-Kopeć et al. [51] confirmed the prevalence of three genes (*YPS2*, *YPS4*, and *YPS6*) in the majority of *C. glabrata* strains isolated from clinical specimens.

Casein is a mixture of phosphoproteins that can be hydrolyzed by a series of enzymes collectively called caseinases. These enzymes belong most likely to the metallo and serine protease families [52]. Caseinase activity was not detected under the employed experimental conditions for any of the tested *C. glabrata* strains. However, these results were discordant from those found by Abbes et al. [53] who reported caseinase activity in 16 *C. glabrata* isolates. Secretion of caseinase has also been observed in *Candida parapsilosis* sensu stricto [24], *Candida haemulonii* species complex [54], and *Yarrowia lipolytica* [53]. Pärnänen et al. [55] identified a serine protease in *C. glabrata* linked to the fungal cell wall, but its role in virulence of *C. glabrata* remains uncertain.

Phospholipases and esterases are extracellular lipolytic enzymes involved in virulence of *Candida* spp. [24, 54]. Their possible functions include digestion of lipids for nutrient acquisition, adhesion to cells and tissues of the host, synergistic interactions with other enzymes, nonspecific hydrolysis, initiation of inflammatory processes by affecting cells of the immune system, and self-defense [56]. In this work, none of the *C. glabrata* strains had detectable levels of phospholipase. Udayalaxmi et al. [57] also did not find phospholipase activity in 14 *C. glabrata* clinical strains isolated from the genitourinary tract. A study from Brazil detected phospholipase activity by the agar plate methodology only in one *C. glabrata* strain isolated from the nasolacrimal duct outlet of a horse [58], thus confirming the low phospholipase production in *C. glabrata*, especially those isolated from human clinical specimens. In a survey among *Candida* vaginal isolates from Egypt, phospholipase activity was observed in a small number of *C. glabrata* strains. This same study also detected the phospholipase *PB2* gene in a few strains studied. On the other hand, the incidence of the phospholipase *PB1* gene in the *Candida* population studied was high, ranging from 87.5% to 95%, depending on the patient history for diabetes [59].

Esterase production was the virulence-related phenotype with more variation among the strains of this study. In a study from Iran with eight *C. glabrata* strains isolated from the oral mucosa, the esterase production showed less variation than the present work, with most strains classified as esterase producers [60]. On the other hand, a study from Turkey revealed that only one from 14 *C. glabrata* strains isolated from bloodstream infection was considered positive in the esterase agar assay. These data suggest that esterase production in *C. glabrata* may be highly heterogeneous according to the source of the clinical material or the geographic region from which the strains were isolated. A major production of esterase was observed in MCF susceptible *C. glabrata* strains. Enzymes with the ability to degrade chitin are also classified as esterases [61], and high chitin levels are associated with a resistance to caspofungin in some *Candida* species [62]. We are unaware to what extent the esterase agar plate assay employed in this study can also detect chitin desacetylases or if the expression of genes for all esterase families has the same regulation in *C. glabrata* strains. The Spearman's correlation analysis revealed that as MCF MIC increases, esterase production does not increase, which could be the reflex of a higher chitin content in the cell walls of resistant strains due to a lower chitin degradation. Further studies are under way to check this hypothesis.

Phytase is a phosphohydrolase that cleaves phytate-releasing inorganic phosphate and inositol, two essential nutrients for all living cells [63]. In this study, phytase activity was detected in all *C. glabrata* strains. Similar results have been reported in different *Candida* spp., including *C. glabrata* [26], *Candida parapsilosis* species complex [24, 64], and *Candida haemulonii* species complex [54]. In *Candida* spp., the maintenance of a supply of inositol and phosphate mediated by phytase seems to be especially important for pathogen survival and persistence in the host [26]. It was observed that as AMB MIC increases, phytase production does not decrease in the *C. glabrata* strains of our study. To the best of our knowledge, there are no reports of a correlation between phytase production and AMB MIC. Although we were not able to find differences between median phytase *Pz* values among susceptible and resistant AMB strains, the *P* value obtained by the Mann–Whitney test was low (*P* = 0.07), and the difference observed between the two statistic tests may be explained by the low number of AMB-resistant strains in the studied population.

Iron uptake is one of the fundamental requirements for pathogenic fungi to survive and grow into their hosts. Therefore, their survival depends on specialized mechanisms in order to adapt to the restrictions of micronutrients during pathogenesis. In general, fungi have to lyse red blood cells to assimilate the iron associated with hemoglobin [65]. Only one of the *C. glabrata* strains of this study was unable to produce hemolysins, results that agree with previous publications [66–68], reflecting the importance of this virulence factor for this yeast. In fact, iron uptake mechanisms have been demonstrated as necessary for virulence in *C. glabrata* [69]. Iron uptake is also involved in resistance of *Cryptococcus neoformans* [70] and *Candida* species [71] to FLC. During FLC resistance acquirement by a *C. glabrata* strain exposed to crescent concentrations of this azole, an enhancement of hemolytic activity associated with an overexpression of the hemolysin gene was also observed [72]. Therefore, we would expect that azole-resistant strains would express more hemolysins. Since low numbers of FLC and ITC cross-resistance were observed in our study, we could speculate that the different iron-dependent mechanisms regulate resistance to the different azoles. Surprisingly, it was also noticed that expressions of hemolysins were higher in *C. glabrata* strains resistant to MCF. A synergistic effect between MCF and deferasirox, an iron chelator, has been described for *Pythium insidiosum*, suggesting that iron enhances resistance to this echinocandin [73]. Our results support that a similar mechanism occurs in *C. glabrata*. In sum, iron uptake is associated not only to azole resistance in *C. glabrata*, but also to the resistance to echinocandin drugs, such as MCF. These results encourage the development of new therapeutic strategies involving iron depletion, already described for *C. albicans* [74], for the treatment of invasive *C. glabrata* infections.

Catalase was expressed by all tested strains. However, no correlation was observed between the activity of this enzyme and the antifungal susceptibility of these clinical isolates. *C. glabrata* possesses both enzymatic and glutathione mechanisms to resist to the oxidative stress induced by the host immune defenses [75], and our results reinforce the importance of enzymatic mechanisms to maintain redox homeostasis in clinical *C. glabrata* strains.

## 5. Conclusions

These findings contribute to a better understanding of the *C. glabrata* pathogenesis, showing that aspartic protease, esterase, phytase, hemolysin, and catalase are present in strains from clinical origin. Moreover, the association between expression of some virulence factors with the antifungal resistance to polyenes, azoles, and echinocandins encourages the development of new therapeutic synergistic strategies involving virulence mechanisms such as hydrolytic enzymes as potential targets against drug resistance in *C. glabrata* infections.

## Figures and Tables

**Table 1:** In vitro antifungal susceptibility profile in 91 Brazilian clinical strains of *Candida glabrata.*

Antifungals	MIC (*μ*g/mL)			CLSI interpretation Number of strains (%)		
	Range	MIC_50_/MIC_90_	GM	S	SDD or I	R
Amphotericin B^1^	0.06–8	0.5/2	0.61	82 (90.1)	—	9 (9.9)
Fluconazole^2^	0.5–≥64	16/64	11.23	—	77 (84.6)	14 (15.4)
Itraconazole^1^	0.016–4	0.25/0.5	0.22	31 (34.1)	55 (60.4)	5 (5.5)
Micafungin^2^	0.016–1	0.06/0.25	0.08	51 (56.0)	26 (28.6)	14 (15.4)
5-Flucytosine^1^	0.12	0.12/0.12	0.12	91 (100.0)	—	—

MIC: minimal inhibitory concentration; CLSI: clinical and laboratory standards institute; GM: geometric mean; S: susceptible; SDD: susceptible-dose dependent; I: intermediary; R: resistant. ^1^Breakpoints established by M27-S3 protocol [20]. In sum, strains with amphotericin B MIC > 1 *μ*g/mL are likely to be resistant to this drug; itraconazole MIC ≤ 0.125 *μ*g/mL are likely to be susceptible, 0.25 ≤ MIC ≤ 0.5 *μ*g/mL are likely to be intermediary, and MIC ≥ 1 *μ*g/mL are likely to be resistant to this drug; 5-flucytosine MIC ≤ 4 *μ*g/mL are likely to be susceptible, 8 ≤ MIC ≤ 16 *μ*g/mL are likely to be intermediary, and MIC ≥ 32 *μ*g/mL are likely to be resistant to this drug. ^2^Breakpoints established by M27-S4 protocol [21]. In sum, *C. glabrata* strains with fluconazole MIC ≤ 32 *μ*g/mL are likely to be susceptible-dose dependent and MIC ≥ 64 *μ*g/mL are likely to be resistant to this drug; *C. glabrata* strains with micafungin MIC ≤ 0.06 *μ*g/mL are likely to be susceptible, MIC = 0.12 *μ*g/mL are likely to be intermediary, and MIC ≥ 0.25 *μ*g/mL are likely to be resistant to this drug.

**Table 2:** Resistance to at least two antifungal drugs in Brazilian clinical strains of *Candida glabrata.*

Antifungals	Number of strains (%)	Clinical specimen (number of strains)
AMB and FLC	5 (5.5)	Bronchoalveolar lavage (1), pleural fluid (1), blood (2), vaginal secretion (1)
AMB, FLC, and MCF	1 (1.1)	Feces (1)
FLC and ITC	2 (2.2)	Sputum (1), urine (1)
FLC and MCF	2 (2.2)	Blood (1), vaginal secretion (1)
FLC, ITC, and MCF	1 (1.1)	Vaginal secretion (1)

AMB: amphotericin B; FLC: fluconazole; ITC: itraconazole; MCF: micafungin.

**Table 3:** Correlation between production of five potential fungal virulence-related enzymes and minimum inhibitory concentrations of four different antifungal drugs in 91 Brazilian clinical strains of *Candida glabrata.*

Antifungals	Hydrolytic enzymes, *P*^∗^ (*r_s_*)				
	Aspartic protease	Esterase	Phytase	Hemolysin	Catalase
Amphotericin B	0.7409 (0.04)	0.3865 (0.09)	**0.0353 (−0.22)**	0.1771 (−0.14)	0.2567 (0.12)
Fluconazole	0.7230 (−0.04)	0.1493 (0.15)	0.0910 (−0.18)	**0.0040 (−0.30)**	0.4128 (−0.09)
Itraconazole	0.6495 (0.05)	0.4667 (0.08)	0.3749 (0.09)	0.1418 (−0.16)	0.8592 (−0.02)
Micafungin	0.0559 (−0.20)	**<0.0001 (0.40)**	0.3768 (−0.09)	**0.0034 (−0.30)**	0.0922 (−0.18)

^∗^ *P* values of 0.05 or less (in bold) were considered statistically significant.
